# Magnetic techniques for the isolation and purification of proteins and peptides

**DOI:** 10.1186/1477-044X-2-7

**Published:** 2004-11-26

**Authors:** Ivo Safarik, Mirka Safarikova

**Affiliations:** 1 Laboratory of Biochemistry and Microbiology, Institute of Landscape Ecology, Academy of Sciences, Na Sadkach 7, 370 05 Ceske Budejovice, Czech Republic; 2 Department of General Biology, University of South Bohemia, Branisovska 31, 370 05 Ceske Budejovice, Czech Republic

## Abstract

Isolation and separation of specific molecules is used in almost all areas of biosciences and biotechnology. Diverse procedures can be used to achieve this goal. Recently, increased attention has been paid to the development and application of magnetic separation techniques, which employ small magnetic particles. The purpose of this review paper is to summarize various methodologies, strategies and materials which can be used for the isolation and purification of target proteins and peptides with the help of magnetic field. An extensive list of realised purification procedures documents the efficiency of magnetic separation techniques.

## Introduction

Isolation, separation and purification of various types of proteins and peptides, as well as of other specific molecules, is used in almost all branches of biosciences and biotechnologies. Separation science and technology is thus very important area necessary for further developments in bio-oriented research and technology. New separation techniques, capable of treating dilute solutions or solutions containing only minute amounts of target molecules in the presence of vast amounts of accompanying compounds in both small and large-scale processes, even in the presence of particulate matter, are necessary.

In the area of biosciences and biotechnology the isolation of proteins and peptides is usually performed using variety of chromatography, electrophoretic, ultrafiltration, precipitation and other procedures, affinity chromatography being one of the most important techniques. Affinity ligand techniques represent currently the most powerful tool available to the downstream processing both in term of their selectivity and recovery. The strength of column affinity chromatography has been shown in thousands of successful applications, especially in the laboratory scale. However, the disadvantage of all standard column liquid chromatography procedures is the impossibility of the standard column systems to cope with the samples containing particulate material so they are not suitable for work in early stages of the isolation/purification process where suspended solid and fouling components are present in the sample. In this case magnetic affinity, ion-exchange, hydrophobic or adsorption batch separation processes, applications of magnetically stabilized fluidized beds or magnetically modified two-phase systems have shown their usefulness.

The basic principle of batch magnetic separation is very simple. Magnetic carriers bearing an immobilized affinity or hydrophobic ligand or ion-exchange groups, or magnetic biopolymer particles having affinity to the isolated structure, are mixed with a sample containing target compound(s). Samples may be crude cell lysates, whole blood, plasma, ascites fluid, milk, whey, urine, cultivation media, wastes from food and fermentation industry and many others. Following an incubation period when the target compound(s) bind to the magnetic particles the whole magnetic complex is easily and rapidly removed from the sample using an appropriate magnetic separator. After washing out the contaminants, the isolated target compound(s) can be eluted and used for further work.

Magnetic separation techniques have several advantages in comparison with standard separation procedures. This process is usually very simple, with only a few handling steps. All the steps of the purification procedure can take place in one single test tube or another vessel. There is no need for expensive liquid chromatography systems, centrifuges, filters or other equipment. The separation process can be performed directly in crude samples containing suspended solid material. In some cases (e.g., isolation of intracellular proteins) it is even possible to integrate the disintegration and separation steps and thus shorten the total separation time [1]. Due to the magnetic properties of magnetic adsorbents (and diamagnetic properties of majority of the contaminating molecules and particles present in the treated sample), they can be relatively easily and selectively removed from the sample. In fact, magnetic separation is the only feasible method for recovery of small magnetic particles (diameter ca 0.1 – 1 μm) in the presence of biological debris and other fouling material of similar size. Moreover, the power and efficiency of magnetic separation procedures is especially useful at large-scale operations. The magnetic separation techniques are also the basis of various automated procedures, especially magnetic-particle based immunoassay systems for the determination of a variety of analytes, among them proteins and peptides. Several automated systems for the separation of proteins or nucleic acids have become available recently.

Magnetic separation is usually very gentle to the target proteins or peptides. Even large protein complexes that tend to be broken up by traditional column chromatography techniques may remain intact when using the very gentle magnetic separation procedure [2]. Both the reduced shearing forces and the higher protein concentration throughout the isolation process positively influence the separation process.

Separation of target proteins using standard chromatography techniques often leads to the large volume of diluted protein solution. In this case appropriate magnetic particles can be used for their concentration instead of ultrafiltration, precipitation etc. [3].

The purpose of this review is to summarize various methodologies and strategies which can be employed for the isolation and purification of target proteins and peptides with the help of magnetic materials. An extensive list of realised purification procedures documents the efficiency of magnetic separation techniques. All these information will help the scientists to select the optimal magnetic material and the purification procedure.

## Necessary materials and equipment

The basic equipment for laboratory experiments is very simple. Magnetic carriers with immobilized affinity or hydrophobic ligands, magnetic particles prepared from a biopolymer exhibiting affinity for the target compound(s) or magnetic ion-exchangers are usually used to perform the isolation procedure. Magnetic separators of different types can be used for magnetic separations, but many times cheap strong permanent magnets are equally efficient, especially in preliminary experiments.

**Magnetic carriers and adsorbents **can be either prepared in the laboratory, or commercially available ones can be used. Such carriers are usually available in the form of magnetic particles prepared from various synthetic polymers, biopolymers or porous glass, or magnetic particles based on the inorganic magnetic materials such as surface modified magnetite can be used. Many of the particles behave like superparamagnetic ones responding to an external magnetic field, but not interacting themselves in the absence of magnetic field. This is important due to the fact that magnetic particles can be easily resuspended and remain in suspension for a long time. In most cases, the diameter of the particles differs from ca 50 nm to approx. 10 μm. However, also larger magnetic affinity particles, with the diameters up to millimetre range, have been successfully used [4]. Magnetic particles having the diameter larger than ca 1 μm can be easily separated using simple magnetic separators, while separation of smaller particles (magnetic colloids with the particle size ranging between tens and hundreds of nanometers) may require the usage of high gradient magnetic separators.

Commercially available magnetic particles can be obtained from a variety of companies. In most cases polystyrene is used as a polymer matrix, but carriers based on cellulose, agarose, silica, porous glass or silanized magnetic particles are also available. Examples of magnetic particles used (or usable) for proteins and peptides separation can be found elsewhere [5-7].

Particles with immobilised affinity ligands are available for magnetic affinity adsorption. Streptavidin, antibodies, protein A and Protein G are used most often in the course of protein and peptides isolation. Magnetic particles with above mentioned immobilised ligands can also serve as generic solid phases to which native or modified affinity ligands can be immobilised (e.g., antibodies in the case of immobilised protein A, protein G or secondary antibodies, biotinylated molecules in the case of immobilised streptavidin).

Also some other affinity ligands (e.g., nitrilotriacetic acid, glutathione, trypsin, trypsin inhibitor, gelatine etc.) are already immobilised to commercially available carriers. To immobilise other ligands of interest to both commercial and laboratory made magnetic particles standard procedures used in affinity chromatography can be employed. Usually functional groups available on the surface of magnetic particles such as -COOH, -OH or -NH_2 _are used for immobilisation, in some cases magnetic particles are available already in the activated form (e.g., tosylactivated, epoxyactivated etc).

In the laboratory magnetite (or similar magnetic materials such as maghemite or ferrites) particles can be surface modified by silanization. This process modifies the surface of the inorganic particles so that appropriate functional groups become available, which enable easy immobilisation of affinity ligands [8]. In exceptional cases enzyme activity can be decreased as a result of usage of magnetic particles with exposed iron oxides. In this case encapsulated microspheres, having an outer layer of pure polymer, will be safer.

Biopolymers such as agarose, chitosan, kappa carrageenan and alginate can be easily prepared in a magnetic form. In the simplest way the biopolymer solution is mixed with magnetic particles and after bulk gel formation the magnetic gel formed is mechanically broken into fine particles [9]. Alternatively biopolymer solution containing dispersed magnetite is dropped into a mixed hardening solution [4] or water-in-oil suspension technique is used to prepare spherical particles [10].

Basically the same procedures can be used to prepare magnetic particles from synthetic polymers such as polyacrylamide, poly(vinylalcohol) and many others [11].

In another approach used standard affinity or ion-exchange chromatography material was post-magnetised by interaction of the sorbent with water-based ferrofluid. Magnetic particles accumulated within the pores of chromatography adsorbent thus modifying this material into magnetic form [12,13]. Alternatively magnetic Sepharose or other agarose gels were prepared by simple contact with freshly precipitated or finely powdered magnetite [12,14].

Magnetoliposomes (magnetic derivatives of standard liposomes), either in the original form or after immobilization of specific proteins, have the potential for the separation of antiphospholipid antibodies [15], IgG antibodies [16] and other proteins of interest [17].

Recently also non-spherical magnetic structures, such as magnetic nanorods have been tested as possible adsorbent material for specific separation of target proteins [18].

**Figure 1:**
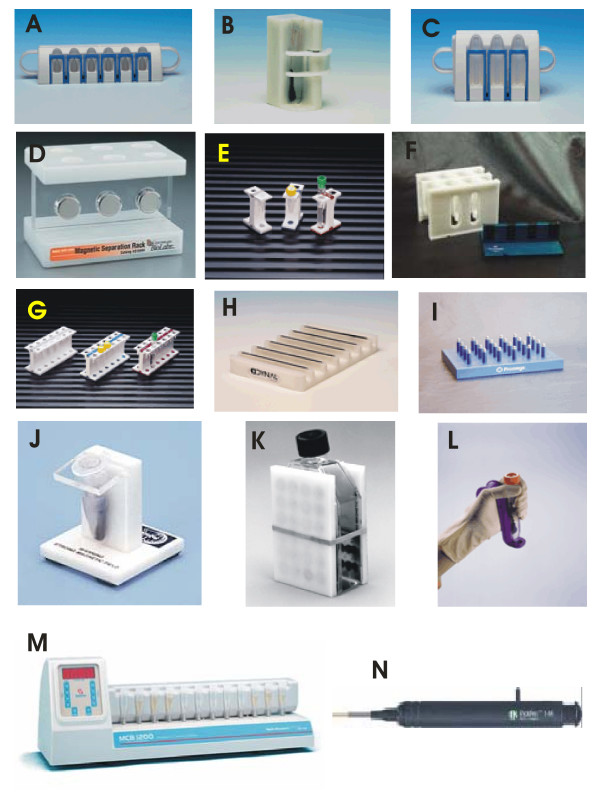
Examples of batch magnetic separators applicable for magnetic separation of proteins and peptides. A: Dynal MPC -S for six microtubes (Dynal, Norway); B: Dynal MPC – 1 for one test tube (Dynal, Norway); C: Dynal MPC – L for six test tubes (Dynal, Norway); D: magnetic separator for six Eppendorf tubes (New England BioLabs, USA); E: MagneSphere Technology Magnetic Separation Stand, two position (Promega, USA); F: MagnaBot Large Volume Magnetic Separation Device (Promega, USA); G: MagneSphere Technology Magnetic Separation Stand, twelve-position (Promega, USA); H: Dynal MPC – 96 S for 96-well microtitre plates (Dynal, Norway); I: MagnaBot 96 Magnetic Separation Device for 96-well microtitre plates (Promega, USA); J: BioMag Solo-Sep Microcentrifuge Tube Separator (Polysciences, USA); K: BioMag Flask Separator (Polysciences, USA); L: MagneSil Magnetic Separation Unit (Promega, USA); M: MCB 1200 processing system for 12 microtubes based on MixSep process (Sigris Research, USA); N: PickPen magnetic tool (Bio-Nobile, Finland). Reproduced with the permission of the above mentioned companies; the photos were taken from their www pages.

**Magnetic separators** are necessary to separate the magnetic particles from the system. In the simplest approach, a small permanent magnet can be used, but various magnetic separators employing strong rare-earth magnets can be obtained at reasonable prices. Commercial laboratory scale batch magnetic separators are usually made from magnets embedded in disinfectant-proof material. The racks are constructed for separations in Eppendorf micro-tubes, standard test tubes or centrifugation cuvettes, some of them have a removable magnetic plate to facilitate easy washing of separated magnetic particles. Other types of separators enable separations from the wells of microtitration plates and the flat magnetic separators are useful for separation from larger volumes of suspensions (up to approx. 500 – 1000 ml). Examples of typical batch magnetic separators are shown in Fig. 1.

Flow-through magnetic separators are usually more expensive, and high gradient magnetic separators (HGMS) are the typical examples. Laboratory scale HGMS is composed from a column packed with fine magnetic grade stainless steel wool or small steel balls which is placed between the poles of an appropriate magnet. The suspension is pumped through the column, and magnetic particles are retained within the matrix. After removal the column from the magnetic field, the particles are retrieved by flow and usually by gentle vibration of the column.

For work in dense suspensions, open gradient magnetic separators may be useful. A very simple experimental set-up for the separation of magnetic affinity adsorbents from litre volumes of suspensions was described [19].

Currently many projects require the analysis of a high number of individual proteins or variants. Therefore, methods are required that allows multiparallel processing of different proteins. There are several multiple systems for high throughput nucleic acid and proteins preparation commercially available. The most often used approach for proteins isolation is based on the isolation and assay of 6xHis-tagged recombinant proteins using magnetic beads with Ni-nitriloacetic acid ligand [20]. The commercially available platforms can be obtained from several companies such as Qiagen, USA (BioRobot and BioSprint series), Tecan, Japan (Te-MagS) or Thermo Electron Corporation, USA (KingFisher).

## Basic principles of magnetic separations of proteins and peptides

Magnetic separations of proteins and peptides are usually convenient and rapid. Nevertheless, several hints may be helpful to obtain good results.

Proteins and peptides in the free form can be directly isolated from different sources. Membrane bound proteins have to be usually solubilized using appropriate detergents. When nuclei are broken during sample preparation, DNA released into the lysate make the sample very viscous. This DNA may be sheared by repeated passage up and down through a 21 gauge hypodermic syringe needle before isolation of a target protein. Alternatively, DNase can be added to enzymatically digest the DNA.

Magnetic beads in many cases exhibit low non-specific binding of non-target molecules present in different samples. Certain samples may still require preclearing to remove molecules which have high non-specific binding activity. If preclearing is needed, the sample can be mixed with magnetic beads not coated with the affinity ligand. In the case of immunomagnetic separation, magnetic beads coated with secondary antibody or with irrelevant antibodies have been used. The non-specific binding can also be minimised by adding a non-ionic detergent both in the sample and in the washing buffers after isolation of the target.

In general, magnetic affinity separations can be performed in two different modes. In the direct method, an appropriate affinity ligand is directly coupled to the magnetic particles or biopolymer exhibiting the affinity towards target compound(s) is used in the course of preparation of magnetic affinity particles. These particles are added to the sample and target compounds then bind to them. In the indirect method the free affinity ligand (in most cases an appropriate antibody) is added to the solution or suspension to enable the interaction with the target compound. The resulting complex is then captured by appropriate magnetic particles. In case antibodies are used as free affinity ligands, magnetic particles with immobilised secondary antibodies, protein A or protein G are used for capturing of the complex. Alternatively the free affinity ligands can be biotinylated and magnetic particles with immobilised streptavidin or avidin are used to capture the complexes formed. In both methods, magnetic particles with isolated target compound(s) are magnetically separated and then a series of washing steps is performed to remove majority of contaminating compounds and particles. The target compounds are then usually eluted, but for specific applications (especially in molecular biology, bioanalytical chemistry or environmental chemistry) they can be used still attached to the particles, such as in the case of polymerase chain reaction, magnetic ELISA etc.

The two methods perform equally well, but, in general, the direct technique is more controllable. The indirect procedure may perform better if affinity ligands have poor affinity for the target compound.

In most cases, magnetic batch adsorption is used to perform the separation step. This approach represents the simplest procedure available, enabling to perform the whole separation in one test-tube or flask. If larger magnetic particles (with diameters above ca 1 μm) are used, simple magnetic separators can be employed. In case magnetic colloids (diameters ranging between tens and hundreds of nanometres) are used as affinity adsorbents, high-gradient magnetic separators have usually to be used to remove the magnetic particles from the system.

Alternatively magnetically stabilised fluidised beds (MSFB), which enable a continuous separation process, can be used. The use of MSFB is an alternative to conventional column operation, such as packed-bed or fluidised bed, especially for large-scale purification of biological products. Magnetic stabilisation enables the expansion of a packed bed without mixing of solid particles. High column efficiency, low pressure drop and elimination of clogging can be reached [21,22].

Also non-magnetic chromatographic adsorbents can be stabilized in magnetically stabilized fluidized beds if sufficient amount of magnetically susceptible particles is also present. The minimum amount of magnetic particles necessary to stabilize the bed is a function of various parameters including the size and density of both particles, the magnetic field strength, and the fluidization velocity. A variety of commercially available affinity, ion-exchange, and adsorptive supports can be used in the bed for continuous separations [23].

Biocompatible two phase systems, composed for example from dextran and polyethylene glycol, are often used for isolation of biologically active compounds, subcellular organelles and cells. One of the disadvantages of this system is the slow separation of the phases when large amounts of proteins and cellular components are present. The separation of the phases can be accelerated by the addition of fine magnetic particles or ferrofluids to the system followed by the application of a magnetic field. This method seems to be useful when the two phases have very similar densities, the volumetric ratio between the phases is very high or low, or the systems are viscous. Magnetically enhanced phase separation usually increases the speed of phase separation by a factor of about 10 in well-behaved systems, but it may increase by a factor of many thousands in difficult systems. The addition of ferrofluids and/or iron oxide particles was shown to have usually no influence on enzyme partioning or enzyme activity [24,25].

Proteins and peptides isolated using magnetic techniques have to be usually eluted from the magnetic separation materials. In most cases bound proteins and peptides can be submitted to standard elution methods such as the change of pH, change of ionic strength, use of polarity reducing agents (e.g., dioxane or ethyleneglycol) or the use of deforming eluents containing chaotropic salts. Affinity elution (e.g., elution of glycoproteins from lectin coated magnetic beads by the addition of free sugar) may be both a very efficient and gentle procedure.

## Examples of magnetic separations of proteins and peptides

Magnetic affinity and ion-exchange separations have been successfully used in various areas, such as molecular biology, biochemistry, immunochemistry, enzymology, analytical chemistry, environmental chemistry etc [26-29]. Tables 1, 2, 3, 4, 5, 6, 7, 8, 9 show some selected applications of these techniques for proteins and peptides isolation.

**Table 1:** Examples of proteinases and peptidases purified by magnetic techniques

**Purified enzyme**	**Source**	**Magnetic carrier**	**Affinity ligand**	**Further details**	**Reference**
Aminopeptidase	*Arabidopsis*	Amine-terminated magnetic beads	N-1-Naphthylphthalamic acid	KCl gradient elution	[54]
Angiotensin-converting enzyme	Pig lung membranes	Dynabeads	Polyclonal antibodies		[57]
Bromelain	Commercial preparation	Polyacrylic acid – iron oxide magnetic nanoparticles		Elution with KCl solution	[59]
Caspase (recombinant, histidine-tailed)	Human cells	Magnetic agarose	Ni-NTA	Elution with SDS-PAGE buffer	[60]
Chymotrypsin	Commercial preparation	Magnetic chitosan beads		Elution with N-acetyl-D-tryptophan	[62]
NIa-protease (recombinant, histidine-tagged)	Plum Pox Virus	Magnetic core and nickel-silica composite matrix	Ni^2+^	Elution with imidazole containing buffer	[36]
Proteinases	Commercial sources	Magnetic cross-linked erythrocytes		Elution with low pH buffer	[46]
Proteinase, bacterial (Savinase)	*Bacillus clausii*	Silanized magnetite particles	Bacitracin		[84]
Trypsin	Porcine pancreatin	Silanized magnetite particles	p-Aminobenzamidine	Elution with low pH buffer	[50]
	Porcine pancreatin	Magnetic polymer particles	Soybean trypsin inhibitor	Elution with low pH solution	[86]
	Commercial preparation	Silanized ferrite powder	Soybean trypsin inhibitor		[87]
	Commercial preparation	Magnetic κ-carrageenan particles	Soybean trypsin inhibitor	Elution with low pH solution, MSFB	[88][89]
	Commercial preparation	Magnetic polyacrylamide beads	Soybean trypsin inhibitor	Magnetically stabilized fluidized beds	[90]
	Commercial preparation	Magnetic chitosan particles	Aprotinin	Elution with low pH solution	[91]
	Commercial preparation	Magnetic cross-linked erythrocytes		Elution with low pH buffer; separation from large volume sample	[19]
Urokinase	Crude urokinase preparation	Magnetic dextran, agarose, polyvinyl alcohol, polyhydroxyethyl methacrylate microspheres	p-Aminobenzamide, L-arginine methyl ester, guanidine hexanoic acid or guanidine acetic acid		[93]

**Table 2:** Purification of lysozyme by magnetic techniques

**Purified enzyme**	**Source**	**Magnetic carrier**	**Affinity ligand**	**Further details**	**Reference**
Lysozyme	Hen egg white	Magnetic chitin		Elution with 0.01 M HCl	[71]
	Hen egg white	Magnetic acetylated chitosan		Elution with 0.01 M HCl	[9]
	Commercial preparation	Magnetic poly(2-hydroxyethyl methacrylate)	Cibacron Blue F3GA	Elution with 1 M KSCN	[72]
		Magnetic chitosan beads		Magnetically stabilized fluidized bed	[73]
	*Ornithodoros moubata*	Magnetic chitin		Elution with alkaline, high salt buffer	[74]
	Commercial preparation	Magnetic cross-linked polyvinylalcohol	Cibacron blue 3GA	Elution with high salt buffer	[52]
		Magnetite – polyacrylic acid nanoparticles		Ion-exchange separation	[75]
		Magnetic cross-linked polyvinylalcohol beads		Adsorption study	[76]
	Commercial preparation	Magnetic agarose beads	Cibacron blue 3GA	Magnetically stabilized fluidized bed	[77]
		Magnetic chitosan	Cibacron blue 3GA	Study of adsorption properties	[78]
	Commercial preparation	Ferrofluid modified sawdust		Elution with 0.5 M NaCl	[79]
	Commercial preparation	Nano-sized magnetic particles		Elution with NaH_2_PO_4 _and NaSCN	[80]
Lysozyme (recombinant, histidine-tailed)	T4	BioMag, amine terminated	Iminodiacetic acid charged with Cu^2+^	Elution with low pH buffer	[81]

**Table 3:** Examples of polysaccharide and disaccharide hydrolases purified by magnetic techniques

**Purified enzyme**	**Source**	**Magnetic carrier**	**Affinity ligand**	**Further details**	**Reference**
α-Amylases	Porcine pancreas,* Bacillus subtilis*, wheat germ	Magnetic alginate beads		Elution with 1 M maltose	[4]
	*Bacillus amyloliquefaciens*, porcine pancreas	Magnetic alginate microbeads		Elution with 1 M maltose	[10]
β-Amylase	Sweet potato	Magnetic alginate beads		Elution with 1 M maltose	[55]
β-Galactosidase	*Escherichia coli *homogenate	Silanized magnetite	p-Aminophenyl-β-D-thiogalactopyranoside	Elution with borate buffer, pH 10	[58]
β-Galactosidase (fusion protein comprising the DNA-binding lac repressor)	Bacterial lysate	Magnetic beads	DNA containing *Escherichia coli *lac operator	Elution with lactose analogue	[64]
Glucoamylase	*Aspergillus niger*	Magnetic alginate beads		Elution with 1 M maltose	[55]
Pectinase	Commercial preparation	Magnetic alginate beads			[82]
Pullulanase	*Bacillus acidopullulyticus*	Magnetic alginate beads		Elution with 1 M maltose	[55]

**Table 4:** Examples of other enzymes purified by magnetic techniques

**Purified enzyme**	**Source**	**Magnetic carrier**	**Affinity ligand**	**Further details**	**Reference**
Alcohol dehydrogenase	Yeast homogenate	Magnetic cross-linked polyvinylalcohol	Cibacron blue 3GA	Elution with high salt buffer	[52]
	*Saccharomyces cerevisiae *extract		PEG with bound Cibacron blue	Magnetic two-phase system	[53]
Aldolase (recombinant, histidine tagged)	Pea	Magnetic core and nickel-silica composite matrix	Ni^2+^	Elution with imidazole containing buffer	[36]
AngioI-TEM-β-lactamase	*Escherichia coli *cells extracts	Magnetic agarose beads	Iminodiacetic acid charged with Zn^2+^	Elution with low pH buffer	[56]
Asparaginase	*Escherichia coli *homogenate	Magnetic polyacrylamide gel particles	D-Asparagine	Elution with D-asparagine solution	[58]
Carbonic anhydrase	Model mixture	Magnetic agarose beads	Sulfanilimide	Elution with high salt buffer	[14]
Catalase	Bovine liver, commercial preparation	Magnetic poly(EGDMA-MAH) beads	Fe^3+^	Elution with NaSCN solution	[61]
Cytochrome c	Horse, *Candida krusei*	Amine terminated iron oxide particles	Iminodiacetic acid charged with Cu^2+^	Binding studies	[63]
	Commercial preparation	Au@magnetic particles		MALDI MS analysis	[31]
	Horse heart	Magnetic agarose beads	Iminodiacetic acid charged with Cu^2+^	Elution with EDTA containing buffer	[56]
	Bovine heart	Magnetic ion-exchange particles		Protein binding studies	[12]
Glucose-6-phosphate dehydrogenase		Ferrofluid modified Sepharose 4B	ADP		[65]
	*Saccharomyces cerevisiae *extract		PEG with bound Cibacron blue	Magnetic two-phase system	[53]
Hexokinase	*Escherichia coli *homogenate		PEG with bound Cibacron blue	Magnetic two-phase system	[53]
Lactate dehydrogenase	Beef heart	Ferrofluid modified Sepharose 4B	AMP	Elution with 1 mM NADH	[13]
	Porcine muscle	Magnetic agarose beads	Reactive Red 120	Column elution with NaCl gradient	[66]
Lactoperoxidase	Sweet whey	Magnetic cation exchanger		HGMS	[67,68]
Luciferase (histidine-tagged)	*Escherichia coli *homogenate	MagneHis™ system	Ni^2+^		[69,70]
Phosphatase, alkaline	Human placenta	Dynabeads M-450	Specific antibody	Activity of bound enzyme measured	[83]
Phosphatase, alkaline (fusion protein comprising the DNA-binding lac repressor)	Bacterial lysate	Magnetic beads	DNA containing *Escherichia coli *lac operator	Elution with lactose analogue	[64]
Phosphofructokinase	*Saccharomyces cerevisiae *extract		PEG with bound Cibacron blue	Magnetic two-phase system	[53]
6-Phosphogluconate dehydrogenase		Ferrofluid modified Sepharose 4B	ADP	Elution with 1 mM NADP	[13]
Thioredoxin (recombinant, histidine-tagged)	*Escherichia coli*	Magnetic agarose	Ni-NTA	Elution with imidazole containing buffer	[20]
tRNA methionyl synthetase (recombinant, histidine-tagged)	*Escherichia coli*	MagneHis™ system	Ni^2+^	Rapid detection and quantitation of isolated protein	[85]
Uricase (recombinant, histidine-tailed)	*Bacillus*	Ion-chelating magnetic agarose beads	Ni^2+^	Elution by cleavage with proteinase K	[92]

**Table 5:** Examples of antibodies purified by magnetic techniques

**Purified antibody**	**Source**	**Magnetic carrier**	**Affinity ligand**	**Further details**	**Reference**
Anti-BODIPY-fluorescein antibodies		Magnetoliposomes	BODIPY-fluorescein		[94]
Anti-DNA antibody	Systemic lupus erythematosus patient plasma	Magnetic poly(2-hydroxyethyl-methacrylate) beads	DNA	Desorption with 1 M NaSCN solution	[95]
Anti-human chorionic gonadotropin antibody	Murine ascites supernatants	Magnetic cellulose beads	Human chorionic gonadotropin		[96]
Antibody (from rat)	Sample from affinity chromatography	Dynabeads M-280	Sheep anti-rabbit IgG	Antibody concentration	[3]
Antibody	Rabbit serum	Dynabeads M-280	Sheep anti-rabbit IgG	Elution with 0.5 M acetic acid	[97]
Monoclonal antibodies	Mouse hybridoma culture broth	Magnetite particles	Protein A		[98]
Anti-bovine serum albumin antibodies		Thermosensitive magnetic microspheres	Bovine serum albumin	Immobilization by the carbodiimide method	[99]
Immunoglobulin G, human	Commercial preparation	Magnetic poly(ethylene glycol dimethacrylate-N-methacryloly-L-histidine-methylester) beads		Elution with 1 M NaCl	[100]
Immunoglobulin G	Blood serum	Carboxyl-terminated magnetic particles	MproteinAG		[101]
IgE antibodies	Allergic patients sera	Magnetoliposomes	Antigenic proteins		[16]
Murine anti-fibroblast growth factor receptor 1 IgM	Ascites	Polystyrene magnetic beads	Rat anti-mouse IgM monoclonal antibody		[102]

**Table 6:** Examples of DNA/RNA/oligonucleotide/aptamer binding proteins purified by magnetic techniques

**Purified protein**	**Source**	**Magnetic carrier**	**Affinity ligand**	**Further details**	**Reference**
CUG binding proteins	Human myoblasts or fibroblasts	Dynabeads M-280 streptavidin	Biotinylated(CUG)_10_	Elution with 1 M NaCl	[103]
Transcription factor τ	*Saccharomyces cerevisiae*	Dynabeads M-280 streptavidin	Biotinylated tRNA^Glu ^gene fragment	Elution with high salt buffer	[104,105]
DNA-binding proteins	Crude tissue extract	Magnetic phospho cellulose particles			[106]
DNA-binding proteins	*Escherichia coli*	Magnetic phospho cellulose particles			[107]
DNA-binding proteins	HeLa nuclear extracts	Dynabeads M-280 streptavidin	Biotin-labelled DNA fragment	Elution with 2 M NaCl	[108]
*Vaccinia *virus early transcription factor	*Vaccinia *virions	Dynabeads M-280 streptavidin	Biotinylated double-stranded DNA	Elution with high salt buffer	[109]
Ecdysteroid receptor	*Drosophila melanogaster *nuclear extract	Dynabeads M-280 streptavidin	Biotinylated double-stranded oligonucleotide	Elution with 0.4 M KCl	[110]
NanR protein (recombinant)	*Escherichia coli*	μMACS streptavidin MicroBeads	Biotin-labelled DNA fragment	Elution with 1 M NaCl	[111]
p27	Rabbit hepatocytes	Dynabeads M-280 streptavidin	Guanine-rich single-stranded DNA	Elution with NaCl solution	[112]
Pigpen protein	Endothelial cells	Magnetic streptavidin beads	Biotinylated aptamer	Elution with 1 M NaCl	[113]
RNA binding proteins	*Saccharomyces cerevisiae*	μMACS streptavidin MicroBeads	Biotin-labelled RNA probe	Elution with 1 M NaCl	[114]
Single-stranded telomere binding protein (sTBP)	Nuclei from vertebrate tissues	Dynabeads M-280 streptavidin	Biotinylated single stranded TTAGGG_n _repeats	Elution with high salt buffer	[115]
Transcription proteins	Human myeloid cells	Dynabeads M-280 streptavidin	Biotinylated serum inducible element (hSIE)	Elution with high salt buffer	[116]
Transcription factor γRF-1	Human monocytes and epidermal cells	Dynabeads M-280 streptavidin	Biotinylated DNA containing γRF-1 sequences	Elution with 0.6 M KCl	[117]
Protein factor MS2	Murine skeletal myotubes	Dynabeads	Double-stranded DNA	Elution with 100 mM sodium acetate, pH 4.2	[118]
Guide RNA binding protein	*Trypanosoma brucei *mitochondria	Dynabeads M-450 goat anti-mouse IgG	Monoclonal antibody	Elution with low pH buffer cont. SDS	[119]
RNA binding proteins	Pollen grains	Streptavidin MagneSphere particles	Biotinylated oligonucleotides	Elution with SDS buffer	[120]
DNA binding protein	*Schistosoma mansoni*	Dynabeads M-280 streptavidin	Biotinylated DNA	Elution with sodium acetate buffer	[121]
ssDNA binding proteins	Transfected mouse fibroblasts	Dynabeads anti-rabbit IgG	Rabbit antibody	Indirect method	[122]
Tenascin-C	Glioblastoma cells	Dynabeads streptavidin	Biotinylated aptamer	Elution with high salt buffer	[123]
Thermostable brain factor (ThBF)	Rat brain	Streptavidin magnetic particles	Biotinylated oligonucleotides	Elution with 0.7 M KCl	[124]
TTF1 protein	*Escherichia coli *lysate	Dynabeads M-280 streptavidin	Biotinylated aptamer	Elution with DNase	[125]

**Table 7:** Purification of albumin and haemoglobin by magnetic techniques

**Purified protein**	**Source**	**Magnetic carrier**	**Affinity ligand**	**Further details**	**Reference**
Albumin, bovine serum	Commercial preparation	Magnetic agar beads	Cibacron blue3GA	Adsorption experiments	[126]
	Commercial preparation	Magnetic cross-linked polyvinylalcohol	Cibacron blue3GA	Adsorption experiments	[76,127]
		Magnetic chitosan microspheres	Cibacron blue3GA		[78]
	Commercial preparation	Magnetic poly(glycidyl methacrylate-triallyl isocyanurate-divinylbenzene) particles		Anion exchange separation	[128]
	Commercial preparation	Magnetic poly(ethylene glycol dimethacrylate-co-N-methacryloyl-(L)-histidine methyl ester) microbeads	Cu^2+^	Elution with 1.0 M NaSCN	[129]
Albumin, human serum	Commercial preparation	Magnetic poly(2-hydroxyethylmethacrylate) beads	Iminodiacetic acid charged with Cu^2+^	Elution with 1.0 M NaSCN	[130]
	Human plasma	Magnetic poly(2-hydroxyethyl methacrylate) beads	Cibacron blue F3GA	Elution with 0.5 M NaSCN	[131]
	Commercial preparation	Magnetic particles covered with thermosensitive polymer	-	Desorption by decreasing temperature	[132,133]
Albumin, human serum (recombinant, FLAG tagged)	Yeast cells	Magnetic glass beads	Anti-FLAG antibody	Elution with EDTA containing buffer	[1]
Glycated haemoglobin	Human blood	Magnetic poly(vinyl alcohol) beads	m-Aminophenyl-boronic acid	Elution with sorbitol	[138]
Haemoglobin	Bovine, commercial preparation	Amine terminated iron oxide particles	Iminodiacetic acid charged with Cu^2+^	Elution with imidazole containing buffer	[63]
Haemoglobin A1c	Human blood	Magnetic particles isolated from *Magnetospirillum magneticum *AMB-1	*m*-Aminophenyl-boronic acid	used for affinity immunoassay	[150]

**Table 8:** Examples of other proteins purified by magnetic techniques

**Purified protein**	**Source**	**Magnetic carrier**	**Affinity ligand**	**Further details**	**Reference**
Aprotinin	Bovine pancreatic powder	Magnetic chitosan particles	Trypsin	Elution with low pH buffer	[134]
Concanavalin A	Jack bean extract	Magnetic particles	Dextran		[68,135]
*Solanum tuberosum *lectin	Potato tuber	Magnetic chitosan		Elution with low pH buffer	[136]
Green fluorescent protein (histidine tagged)		Magnetic nanoparticles	Ni-NTA	Elution with imidazole containing buffer	[137]
SIRT2 protein (recombinant, histidine tailed)	Human	Magnetic agarose beads	Ni-NTA	Elution with imidazole containing buffer	[139]
Elongation factor (recombinant, histidine tailed)	*Caenorhabditis elegans*	Magnetic agarose beads	Ni-NTA	Elution with imidazole containing buffer	[140]
Protein A	Recombinant *Escherichia coli*	Magnetic Eudragit	Human IgG	Magnetic two-phase system	[141]
Tumor necrosis factor (TNF)		Dynabeads M-280	Mouse monoclonal antibody	Solid phase immunoassay	[142]
Anti-MUC1 diabody fragment	Recombinant *Escherichia coli*	Magnetic agarose beads	Specific peptide		[143]
MHC class II molecules	MDCK cells	Dynabeads M-450 rat anti-mouse IgG1	Specific antibodies	Elution with SDS-PAGE buffer	[144]
Lamin B_3_	*Xenopus *egg extracts	Dynabeads	Specific antibodies	Elution with 6 M urea	[44]
6x-His-tagged proteins	Human fibroblasts	Magnetic agarose beads	Ni-NTA	Elution with imidazole containing buffer	[145]
Estrogen receptor	Adipose tissue	Dynabeads M-280 streptavidin	Biotinylated monoclonal mouse anti-human estrogen receptor antibody	Indirect method	[146]
Thiol-reactive chromatin restriction fragments	Mouse fibroblasts	Mercurated agarose magnetic beads	p-Hydroxymercuribenzoate	Elution with 0.5 M NaCl and 20 mM dithiothreitol	[147]
L1 coat protein	Human papillomavirus	Magnetic polyglutaraldehyde particles	Iminodiacetic acid charged with Cu^2+^	Elution with imidazole containing buffer	[41]
Insulin receptor	Rat muscle or liver extract	Dynabeads M-450	Anti-P5 antibody	SDS PAGE analysis	[148]
Stat3	DER cells	Dynabeads	Biotinylated tyrosine phosphorylated peptides	SDS PAGE analysis	[149]
Transferrin receptor	Human	Dynabeads M-450 sheep anti-mouse IgG1	Anti-human transferrin receptor monoclonal antibody	SDS analysis	[151]
Prion protein PrP^Sc^	Brain extract	Dynabeads M-280 tosyl activated	Plasminogen	SDS analysis	[39,40]
Biotinylated proteins from extracellular matrix	*Bipolaris sorokiniana*	Dynabeads	Streptavidin	SDS analysis	[152]
Cryoprotectin	Leaves of cold-acclimated cabbage (*Brassica oleracea*)	Dynabeads-protein A	Specific antibody		[153]
Prostate specific antigen	Serum from a prostate cancer suffering patient	Streptavidin-coated magnetic beads	Biotinylated monoclonal antibody	Elution with low pH solution	[154,155]
Estrogen receptor	In vitro translation	Magnetic beads	Antibody	Elution with SDS buffer	[156]
VHDL receptor	*Helicoverpa zea*	Streptavidin-coated magnetic beads	VHDL-biotin ligand		[157]
Fructosyllysine-specific binding protein	U937 cells	Dynabeads M-280 tosylactivated	Poly-L-lysine-glucose conjugate	Two proteins isolated	[158]
Ubiquitin (histidine tagged)		Nickel-gold nanorods		Elution with acidic buffer	[18]

**Table 9:** Examples of peptides purified by magnetic techniques

**Purified peptide**	**Source**	**Magnetic carrier**	**Affinity ligand**	**Further details**	**Reference**
Biotinylated peptides	Model mixtures	Dynabeads M-280 streptavidin	Streptavidin	Used in MALDI-TOF mass analysis	[159]
(His)_6_-Ala-Tyr-Gly	Synthetic peptide	Dynabeads M-280 tosylactivated	Aminocaproic nitrilotriacetic acid charged with Ni^2+^	Elution with imidazole solution	[160]
Synthetic pentapeptides against fructose-1,6-biphosphate aldolase	Synthetic mixture	Streptavidin-coated magnetic beads	Biotin labelled fructose-1,6-biphosphate aldolase of *T. brucei*	Pentapeptides were anchored on polystyrene beads	[161]
Tryptic digest products of cytochrome c	Trypsin digested cytochrome c	Au@magnetic particles	-	Ion-exchange separation followed by MALDI MS analysis	[31]
Glutathione		Gold and iron oxide nanocomposites			[162]
Nisin Z	*Lactobacillus lactis*	EDC activated magnetic beads	Anti-nisin antibody	Elution with 6 M urea	[163]

In the case of proteins and peptides purifications, no simple strategy for magnetic affinity separations exists. Various affinity ligands have been immobilised on magnetic particles, or magnetic particles have been prepared from biopolymers exhibiting the affinity for target enzymes or lectins. Immunomagnetic particles, i.e. magnetic particles with immobilised specific antibodies against the target structures, have been used for the isolation of various antigens, both molecules and cells [5] and can thus be used for the separation of specific proteins.

Magnetic separation procedures can be employed in several ways. Preparative isolation of the target protein or peptide is usually necessary if further detailed study is intended. In other cases, however, the magnetic separation can be directly followed (after elution with an appropriate buffer) with SDS electrophoresis. Magnetically separated proteins and peptides can also be used for further mass spectroscopy characterization [30,31]. The basic principles of magnetic separations can be used in the course of protein or peptide determination using various types of solid phase immunoassays. Usually immunomagnetic particles directly capture the target analyte, or magnetic particles with immobilised streptavidin are used to capture the complex of biotinylated primary antibody and the analyte. The separated analyte is then determined (usually without elution) using an appropriate method. A combination of magnetic separation with affinity capillary electrophoresis is also possible [32].

Enzyme isolation is usually performed using immobilised inhibitors, cofactors, dyes or other suitable ligands, or magnetic beads prepared from affinity biopolymers can be used (see Tables 1, 2, 3, 4).

Genetic engineering enables the construction of gene fusions resulting in fusion proteins having the combined properties of the original gene products. To date, a large number of different gene fusion systems, involving fusion partners that range in size from one amino acid to whole proteins, capable of selective interaction with a ligand immobilized onto magnetic particles or chromatography matrices, have been described. In such systems, different types of interactions, such as enzyme-substrate, receptor-target protein, polyhistidines-metal ion, and antibody-antigen, have been utilized. The conditions for purification differ from system to system and the environment tolerated by the target protein is an important factor for deciding which affinity fusion partner to choose. In addition, other factors, including protein localization, costs for the affinity matrix and buffers, and the possibilities of removing the fusion partner by site-specific cleavage, should also be considered [33,34]. As an example, isolation of recombinant oligohistidine-tagged proteins is based on the application of metal chelate magnetic adsorbents [35,36]. This method has been used successfully for the purification of proteins expressed in bacterial, mammalian, and insect systems.

Antibodies from ascites, serum and tissue culture supernatants can be efficiently isolated using magnetic particles with immobilized Protein A, Protein G or anti-immunoglobulin antibodies. Protein A, isolated from *Staphylococcus aureus*, binds the Fc region of IgG of most mammalian species with high affinity, leaving antigen specific sites free. Protein G, isolated from *Streptococcus *sp., reacts with a larger number of IgG isotypes. It has a higher binding affinity to immunoglobulins than Protein A, however, it also interacts with the Fab regions of IgG, although the affinity is ten times lower than for the Fc region [37]. Antiphospholipid antibodies were successfully isolated using magnetoliposomes [15].

Aptamers are DNA or RNA molecules that have been selected from random pools based on their ability to bind other molecules. Aptamers binding proteins can be immobilised to magnetic particles and used for isolation of target proteins.

DNA/RNA binding proteins (e.g., promoters, gene regulatory proteins and transcription factors) are often short-lived and in low abundance. A rapid and sensitive method, based on the immobilization of biotinylated DNA/RNA fragments containing the specific binding sequence to the magnetic streptavidin particles, can be used. The bound DNA/RNA binding proteins are usually eluted with high salt buffer or change of pH [38].

Other types of proteins were isolated using specific affinity-based procedures. For example, plasminogen immobilized on magnetic particles was used to separate scrapie and bovine spongiform encephalopathy associated prion protein PrP^Sc ^from its conformer which is a cellular protein called PrP^C^. In fact, plasminogen represents the first endogenous factor discriminating between normal and pathological prion protein. This unexpected property may be exploited for diagnostic purposes [39,40].

Magnetic separation was also successfully used for the recovery of proteins expressed in the form of inclusion bodies, involving at first chemical extraction from the host cells, then adsorptive capture of the target protein onto small magnetic adsorbents, followed by rapid collection of the product-loaded supports with the aid of high gradient magnetic fields [41].

A new approach for analytical ion-exchange separation of native proteins and proteins enzymatic digest products has been described recently [31]. Magnetite particles were covered with a gold layer and then stabilized with ionic agents. These charged stabilizers present at the surface of the gold particles are capable of attracting oppositely charged species from a sample solution through electrostatic interactions. Au@magnetic particles having negatively charged surfaces are suitable probes for selectively trapping positively charged proteins and peptides from aqueous solutions. The species trapped by the isolated particles were then characterized by matrix-assisted laser desorption/ionization mass spectrometry (MALDI MS) after a simple washing.

Magnetic solid phase extraction (MSPE) enables to preconcentrate target analytes from larger volumes of solutions or suspensions using relatively small amount of magnetic specific adsorbent. Up to now this procedure was used for preconcentration of low-molecular weight xenobiotics [42,43] but using suitable magnetic adsorbents the MSPE could be used to preconcetrate target proteins and peptides as well.

Sometimes the removal of certain proteins will reveal functions involving the depleted proteins or will help in the course of subsequent protein isolation. As an example, Dynabeads have been used to remove involved proteins from *Xenopus *egg extracts for analyses of the cell mitosis mechanisms [44,45]. Rapid removal of contaminating proteolytic enzymes from the crude samples could increase yields of sensitive proteins due to the limitation of their proteolysis [46].

A combination of mechanical cell disintegration and magnetic batch affinity adsorption was used to simplify the isolation of intracellular proteins. Magnetic glass beads were used because of their hardness and rigidity [1].

An example of quite different protein purification strategy can also be mentioned. Proteins associated with the endocytic vesicles of *Dictyostelium discoideum *were separated after magnetic isolation of the vesicles that was accomplished by feeding the amoebae with dextran-stabilized iron oxide particles. The cells were broken, the labelled vesicles were magnetically separated and then disrupted to release proteins which were resolved by SDS-PAGE. After „in-gel“ digestion with endoproteinase Lys-C or Asp-N the generated peptides were used for amino acid sequencing. This strategy allowed the identification of the major protein constituents of the vesicles [47]. Analogous procedure was used for the separation and study of peroxisomes proteins when at first peroxisomes were separated using magnetic beads with immobilized specific antibodies and then the protein content of the separated peroxisomes was analysed [48].

## Conclusions

Standard liquid column chromatography is currently the most often used technique for the isolation and purification of target proteins and peptides. Magnetic separation techniques are relatively new and still under development. Magnetic affinity particles are currently used mainly in molecular biology (especially for nucleic acids separation), cell biology and microbiology (separation of target cells) and as parts of the procedures for the determination of selected analytes using magnetic ELISA and related techniques (especially determination of clinical markers and environmental contaminants). Up to now separations in small scale prevail and thus the full potential of these techniques has not been fully exploited.

It can be expected that further development will be focused at least on two areas. The first one will be focused on the laboratory scale application of magnetic affinity separation techniques in biochemistry and related areas (rapid isolation of a variety of both low- and high-molecular weight substances of various origin directly from crude samples thus reducing the number of purification steps) and in biochemical analysis (application of immunomagnetic particles for separation of target proteins from the mixture followed by their detection using ELISA and related principles). Such a type of analysis will enable to construct portable assay systems enabling e.g. near-patient analysis of various protein disease markers. New methodologies, such as the application of chip and microfluidics technologies, may result in the development of magnetic separation processes capable of magnetic separation and detection of extremely small amount of target biologically active compounds [49].

In the second area, larger-scale (industrial) systems are believed to be developed and used for the isolation of biologically active compounds directly from crude culture media, wastes from food industry etc., integrating three classical steps (clarification, concentration and initial purification) into a single unit operation [50]. It is not expected that extremely large amounts of low cost products will be isolated using magnetic techniques, but the attention should be focused onto the isolation of minor, but highly valuable components present in raw materials. Of course, prices of magnetic carriers have to be lowered and special types of low-cost, biotechnology applicable magnetic carriers and adsorbents prepared by simple and cheap procedures have to become available. The existence of inexpensive and effective magnetic separators enabling large-scale operations is necessary, as well.

In the near future quite new separation strategies can appear. A novel magnetic separation method, which utilizes the magneto-Archimedes levitation, has been described recently and applied to separation of biological materials. By using the feature that the stable levitation position under a magnetic field depends on the density and magnetic susceptibility of materials, it was possible to separate biological materials such as haemoglobin, fibrinogen, cholesterol, and so on. So far, the difference of magnetic properties was not utilized for the separation of biological materials. Magneto-Archimedes separation may be another way for biological materials separation [51].

It can be expected that magnetic separations will be used regularly both in biochemical laboratories and biotechnology industry in the near future.

## References

[B1] Schuster M, Wasserbauer E, Ortner C, Graumann K, Jungbauer A, Hammerschmid F, Werner G (2000). Short cut of protein purification by integration of cell-disrupture and affinity extraction. Bioseparation.

[B2] Hofmann I, Schnolzer M, Kaufmann I, Franke WW (2002). Symplekin, a constitutive protein of karyo- and cytoplasmic particles involved in mRNA biogenesis in *Xenopus laevis *oocytes. Mol Biol Cell.

[B3] Alche JD, Dickinson K (1998). Affinity chromatographic purification of antibodies to a biotinylated fusion protein expressed in *Escherichia coli*. Protein Expr Purif.

[B4] Teotia S, Gupta MN (2001). Purification of alpha-amylases using magnetic alginate beads. Appl Biochem Biotechnol.

[B5] Safarik I, Safarikova M (1999). Use of magnetic techniques for the isolation of cells. J Chromatogr B Biomed Sci Appl.

[B6] Sinclair B (1998). To bead or not to bead: Applications of magnetic bead technology. Scientist.

[B7] Bruce IJ, Taylor J, Todd M, Davies MJ, Borioni E, Sangregorio C, Sen T (2004). Synthesis, characterisation and application of silica-magnetite nanocomposites. J Magn Magn Mater.

[B8] Weetall HH, Lee MJ (1989). Antibodies immobilized on inorganic supports. Appl Biochem Biotechnol.

[B9] Safarik I, Safarikova M (1993). Batch isolation of hen egg white lysozyme with magnetic chitin. J Biochem Biophys Methods.

[B10] Safarikova M, Roy I, Gupta MN, Safarik I (2003). Magnetic alginate microparticles for purification of α-amylases. J Biotechnol.

[B11] Tanyolac D, Ozdural AR (2000). A new low cost magnetic material: magnetic polyvinylbutyral microbeads. React Funct Polym.

[B12] Nixon L, Koval CA, Noble RD, Slaff GS (1992). Preparation and characterization of novel magnetite-coated ion-exchange particles. Chem Mater.

[B13] Mosbach K, Andersson L (1977). Magnetic ferrofluids for preparation of magnetic polymers and their application in affinity chromatography. Nature.

[B14] Hirschbein BL, Whitesides GM (1982). Affinity separation of enzymes from mixtures containing suspended solids. Comparisons of magnetic and nonmagnetic techniques. Appl Biochem Biotechnol.

[B15] Rocha FM, de Pinho SC, Zollner RL, Santana MHA (2001). Preparation and characterization of affinity magnetoliposomes useful for the detection of antiphospholipid antibodies. J Magn Magn Mater.

[B16] Zollner TCA, Zollner RD, de Cuyper M, Santana MHA (2003). Adsorption of isotype "E" antibodies on affinity magnetoliposomes. J Dispersion Sci Technol.

[B17] Bucak S, Jones DA, Laibinis PE, Hatton TA (2003). Protein separations using colloidal magnetic nanoparticles. Biotechnol Progr.

[B18] Lee KB, Park S, Mirkin CA (2004). Multicomponent magnetic nanorods for biomolecular separations. Angew Chem – Int Edit.

[B19] Safarik I, Ptackova L, Safarikova M (2001). Large-scale separation of magnetic bioaffinity adsorbents. Biotechnol Lett.

[B20] Schafer F, Romer U, Emmerlich M, Blumer J, Lubenow H, Steinert K (2002). Automated high-throughput purification of 6xHis-tagged proteins. J Biomol Tech.

[B21] Lochmuller CH, Ronsick CS, Wigman LS (1988). Fluidized-bed separators reviewed: a low pressure drop approach to column chromatography. Prep Chromatogr.

[B22] Burns MA, Graves DJ (1985). Continuous affinity chromatography using a magnetically stabilized fluidized bed. Biotechnol Progr.

[B23] Chetty AS, Burns MA (1991). Continuous protein separations in a magnetically stabilized fluidized bed using nonmagnetic supports. Biotechnol Bioeng.

[B24] Wikstrom P, Flygare S, Grondalen A, Larsson PO (1987). Magnetic aqueous two-phase separation: a new technique to increase rate of phase-separation, using dextran-ferrofluid or larger iron oxide particles. Anal Biochem.

[B25] Larsson P-O (1994). Magnetically enhanced phase separation. Meth Enzymol.

[B26] Safarik I, Safarikova M, Wilson ID, Adlard RR, Poole CF, Cook MR (2000). Biologically active compounds and xenobiotics: Magnetic affinity separations. Encyclopedia of Separation Science.

[B27] Safarik I, Safarikova M, Hafeli U, Schutt W, Teller J, Zborowski M (1997). Overview of magnetic separations used in biochemical and biotechnological applications. Scientific and Clinical Applications of Magnetic Carriers.

[B28] Safarikova M, Safarik I (2001). The application of magnetic techniques in biosciences. Magn Electr Sep.

[B29] Saiyed ZM, Telang SD, Ramchand CN (2003). Application of magnetic techniques in the field of drug discovery and biomedicine. BioMagn Res Technol.

[B30] Yaneva M, Tempst P (2003). Affinity capture of specific DNA-binding proteins for mass spectrometric identification. Anal Chem.

[B31] Teng CH, Ho KC, Lin YS, Chen YC (2004). Gold nanoparticles as selective and concentrating probes for samples in MALDI MS analysis. Anal Chem.

[B32] Heegaard NHH, Nilsson S, Guzman NA (1998). Affinity capillary electrophoresis: important application areas and some recent developments. J Chromatogr B.

[B33] Nilsson J, Stahl S, Lundeberg J, Uhlen M, Nygren PA (1997). Affinity fusion strategies for detection, purification, and immobilization of recombinant proteins. Protein Expr Purif.

[B34] Kobs G (2004). Finding the right protein purification system. Cell Notes.

[B35] Gaberc-Porekar V, Menart V (2001). Perspectives of immobilized-metal affinity chromatography. J Biochem Biophys Methods.

[B36] Frenzel A, Bergemann C, Kohl G, Reinard T (2003). Novel purification system for 6xHis-tagged proteins by magnetic affinity separation. J Chromatogr B.

[B37] Widjojoatmodjo MN, Fluit AC, Torensma R, Verhoef J (1993). Comparison of immunomagnetic beads coated with protein A, protein G, or goat anti-mouse immunoglobulins. J Immunol Methods.

[B38] (1998). Biomagnetic Techniques in Molecular Biology. Information Booklet, Dynal, Oslo, Norway.

[B39] Fischer MB, Roeckl C, Parizek P, Schwarz HP, Aguzzi A (2000). Binding of disease-associated prion protein to plasminogen. Nature.

[B40] Maissen M, Roeckl F, Glatzel M, Goldmann W, Aguzzi A (2001). Plasminogen binds to disease-associated prion protein of multiple species. Lancet.

[B41] Heeboll-Nielsen A, Choe WS, Middelberg APJ, Thomas ORT (2003). Efficient inclusion body processing using chemical extraction and high gradient magnetic fishing. Biotechnol Progr.

[B42] Safarikova M, Safarik I (1999). Magnetic solid-phase extraction. J Magn Magn Mater.

[B43] Safarikova M, Safarik I (2002). Magnetic solid-phase extraction of target analytes from large volumes of urine. Eur Cells Mater.

[B44] Goldberg M, Jenkins H, Allen T, Whitfield WG, Hutchison CJ (1995). Xenopus lamin B3 has a direct role in the assembly of a replication competent nucleus: evidence from cell-free egg extracts. J Cell Sci.

[B45] Bell P, Scheer U (1997). Prenucleolar bodies contain coilin and are assembled in Xenopus egg extract depleted of specific nucleolar proteins and U3 RNA. J Cell Sci.

[B46] Safarik I, Safarikova M (2001). Isolation and removal of proteolytic enzymes with magnetic cross-linked erythrocytes. J Magn Magn Mater.

[B47] Adessi C, Chapel A, Vincon M, Rabilloud T, Klein G, Satre M, Garin J (1995). Identification of major proteins associated with *Dictyostelium discoideum *endocytic vesicles. J Cell Sci.

[B48] Luers GH, Hartig R, Mohr H, Hausmann M, Fahimi HD, Cremer C, Volkl A (1998). Immuno-isolation of highly purified peroxisomes using magnetic beads and continuous immunomagnetic sorting. Electrophoresis.

[B49] Brzeska M, Panhorst M, Kamp PB, Schotter J, Reiss G, Puhler A, Becker A, Bruckl H (2004). Detection and manipulation of biomolecules by magnetic carriers. J Biotechnol.

[B50] Hubbuch JJ, Thomas ORT (2002). High-gradient magnetic affinity separation of trypsin from porcine pancreatin. Biotechnol Bioeng.

[B51] Hirota N, Kurashige M, Iwasaka M, Ikehata M, Uetake H, Takayama T, Nakamura H, Ikezoe Y, Ueno S, Kitazawa K (2004). Magneto-Archimedes separation and its application to the separation of biological materials. Physica B.

[B52] Tong XD, Xue B, Sun Y (2001). A novel magnetic affinity support for protein adsorption and purification. Biotechnol Progr.

[B53] Flygare S, Wikstrom P, Johansson G, Larsson PO (1990). Magnetic aqueous two-phase separation in preparative applications. Enzyme Microb Technol.

[B54] Murphy AS, Hoogner KR, Peer WA, Taiz L (2002). Identification, purification, and molecular cloning of N-1-naphthylphthalmic acid-binding plasma membrane-associated aminopeptidases from Arabidopsis. Plant Physiol.

[B55] Teotia S, Gupta MN (2002). Magnetite-alginate beads for purification of some starch degrading enzymes. Mol Biotechnol.

[B56] Abudiab T, Beitle RR (1998). Preparation of magnetic immobilized metal affinity separation media and its use in the isolation of proteins. J Chromatogr A.

[B57] Barnes K, Murphy LJ, Turner AJ (1994). Immunoseparation of membrane peptidases from pig lung membranes using magnetic beads. Biochem Soc Trans.

[B58] Dunnill P, Lilly MD (1974). Purification of enzymes using magnetic bio-affinity materials. Biotechnol Bioeng.

[B59] Chen D-H, Huang S-H (2004). Fast separation of bromelain by polyacrylic acid-bound iron oxide magnetic nanoparticles. Process Biochem.

[B60] Himeji D, Horiuchi T, Tsukamoto H, Hayashi K, Watanabe T, Harada M (2002). Characterization of caspase-8L: a novel isoform of caspase-8 that behaves as an inhibitor of the caspase cascade. Blood.

[B61] Akgol S, Denizli A (2004). Novel metal-chelate affinity sorbents for reversible use in catalase adsorption. J Mol Catal B – Enzym.

[B62] Ghosh M, Tyagi R, Gupta MN (1995). Preparation of trypsin free chymotrypsin. Biotechnol Tech.

[B63] O'Brien SM, Thomas ORT, Dunnill P (1996). Non-porous magnetic chelator supports for protein recovery by immobilised metal affinity adsorption. J Biotechnol.

[B64] Ljungquist C, Lundeberg J, Rasmussen AM, Hornes E, Uhlen M (1993). Immobilization and recovery of fusion proteins and B-lymphocyte cells using magnetic separation. DNA Cell Biol.

[B65] Griffin T, Mosbach K, Mosbach R (1981). Magnetic biospecific affinity adsorbents for immunoglobulin and enzyme isolation. Appl Biochem Biotechnol.

[B66] Ennis MP, Wisdom GB (1991). A magnetizable solid phase for enzyme extraction. Appl Biochem Biotechnol.

[B67] Justesen SFL, Nielsen AH, Thomas ORT (2001). High gradient magnetic fishing for the isolation of high-value proteins from sweet whey. Danish Biotechnology Conference VII: Vejle, Denmark..

[B68] Heeboll-Nielsen A, Justesen S, Thomas ORT (2001). Product recovery for crude bioprocess liquors by high gradient magnetic fishing. 10th European Congress on Biotechnology: Madrid, Spain..

[B69] Betz N (2004). Efficient purification of His-tagged proteins from insect and mammalian cells. Promega Notes.

[B70] Betz N (2004). Purifying His-tagged proteins from insect and mammalian cells. Cell Notes.

[B71] Safarik I (1991). Magnetic biospecific affinity adsorbents for lysozyme isolation. Biotechnol Tech.

[B72] Odabasi M, Denizli A (2004). Cibacron blue F3GA incorporated magnetic poly(2-hydroxyethyl methacrylate) beads for lysozyme adsorption. J Appl Polym Sci.

[B73] Goto M, Imamura T, Hirose T (1995). Axial dispersion in liquid magnetically stabilized fluidized beds. J Chromatogr A.

[B74] Kopacek P, Vogt R, Jindrak L, Weise C, Safarik I (1999). Purification and characterization of the lysozyme from the gut of the soft tick *Ornithodoros moubata*. Insect Biochem Mol Biol.

[B75] Liao MH, Chen DH (2002). Fast and efficient adsorption/desorption of protein by a novel magnetic nano-adsorbent. Biotechnol Lett.

[B76] Xue B, Sun Y (2001). Protein adsorption equilibria and kinetics to a poly(vinyl alcohol)-based magnetic affinity support. J Chromatogr A.

[B77] Tong XD, Sun Y (2003). Application of magnetic agarose support in liquid magnetically stabilized fluidized bed for protein adsorption. Biotechnol Progr.

[B78] Yu YH, Xue B, Sun Y, He BL (2000). The preparation of chitosan affinity magnetic nanoparticles and their adsorption properties for proteins. Acta Polym Sinica.

[B79] Safarik I, Safarikova M, Weyda F, Mosiniewicz-Szablewska W, Slawska-Waniewska A Ferrofluid-modified plant-based materials as adsorbents for batch separation of selected biologically active compounds and xenobiotics. J Magn Magn Mater.

[B80] Peng ZG, Hidajat K, Uddin MS (2004). Adsorption and desorption of lysozyme on nano-sized magnetic particles and its conformational changes. Colloid Surf B – Biointerfaces.

[B81] O'Brien SM, Sloane RP, Thomas ORT, Dunnill P (1997). Characterisation of non-porous magnetic chelator supports and their use to recover polyhistidine-tailed T4 lysozyme from a crude E. coli extract. J Biotechnol.

[B82] Tyagi R, Gupta MN (1995). Purification and immobilization of *Aspergillus niger *pectinase on magnetic latex beads. Biocatal Biotransform.

[B83] Hendrix PG, Hoylaerts MF, Nouwen EJ, Van de Voorde A, De Broe ME (1992). Magnetic beads in suspension enable a rapid and sensitive immunodetection of human placental alkaline phosphatase. Eur J Clin Chem Clin Biochem.

[B84] Hubbuch JJ, Matthiesen DB, Hobley TJ, Thomas ORT (2001). High gradient magnetic separation versus expanded bed adsorption: a first principle comparison. Bioseparation.

[B85] Engel L, Kar S, Johnson T (2003). Rapid detection and quantitation of His-tagged proteins purified by MagneHis™ Ni-particles. Promega Notes.

[B86] Khng HP, Cunliffe D, Davies S, Turner NA, Vulfson EN (1998). The synthesis of sub-micron magnetic particles and their use for preparative purification of proteins. Biotechnol Bioeng.

[B87] Halling PJ, Dunnill P (1979). Recovery of free enzymes from product liquors by bio-affinity adsorption: Trypsin binding by immobilised soybean inhibitor. European J Appl Microbiol.

[B88] Lochmuller CH, Wigman LS (1987). Affinity separations in magnetically stabilized fluidized beds. Synthesis and performance of packing materials. Sep Sci Technol.

[B89] Lochmuller CH, Wigman LS, Kitchell BS (1987). Aerosol-jet produced, magnetic carrageenan-gel particles: a new affinity chromatography matrix. J Chem Technol Biotechnol.

[B90] Cocker TM, Fee CJ, Evans RA (1997). Preparation of magnetically susceptible polyacrylamide/magnetite beads for use in magnetically stabilized fluidized bed chromatography. Biotechnol Bioeng.

[B91] An XN, Su ZX (2001). Characterization and application of high magnetic property chitosan particles. J Appl Polym Sci.

[B92] Nishiya Y, Hibi T, Oda JL (2002). A purification method of the diagnostic enzyme *Bacillus *uricase using magnetic beads and non-specific protease. Protein Expr Purif.

[B93] Dong YS, Liang F, Jin HX, Xi Q, Chang JH (2002). Preparation of novel magnetic affinity adsorbents and application for purification of urokinase. Chem J Chin Univ – Chin.

[B94] Dumitrascu G, Kumbhar A, Zhou WL, Rosenzweig Z (2001). The use of derivatized magnetoliposomes for extraction of antibodies from aqueous solutions. IEEE Trans Magn.

[B95] Odabasi M, Denizli A (2001). Polyhydroxyethylmethacrylate-based magnetic DNA-affinity beads for anti-DNA antibody removal from systemic lupus erythematosus patient plasma. J Chromatogr B.

[B96] Li X, Li CX, He BL (1998). Preparation and application of magnetic affinity adsorbent based on magnetic cellulose bead. Chem J Chin Univ – Chin.

[B97] Quitadamo IJ, Kostman TA, Schelling ME, Franceschi VR (2000). Magnetic bead purification as a rapid and efficient method for enhanced antibody specificity for plant sample immunoblotting and immunolocalization. Plant Sci.

[B98] Shinkai M, Kamihira M, Honda H, Kobayashi T (1992). Rapid purification of monoclonal antibody with functional magnetite particles. Kag Kog Ronbunshu.

[B99] Kondo A, Kamura H, Higashitani K (1994). Development and application of thermo-sensitive magnetic immunomicrospheres for antibody purification. Appl Microbiol Biotechnol.

[B100] Ozkara S, Akgol S, Canak Y, Denizli A (2004). A novel magnetic adsorbent for immunoglobulin-G purification in a magnetically stabilized fluidized bed. Biotechnol Progress.

[B101] Meng ZH, Lin B, Xie YH, Zhang KQ (2004). Cloning and expression of fused Fc-binding protein (SPA-SPG) and its application in purification of IgG. Progr Biochem Biophys.

[B102] Quitadamo IJ, Schelling ME (1998). Efficient purification of mouse anti-FGF receptor IgM monoclonal antibody by magnetic beads. Hybridoma.

[B103] Bhagwati S, Ghatpande A, Leung B (1996). Identification of two nuclear proteins which bind to RNA CUG repeats: Significance for myotonic dystrophy. Biochem Biophys Res Commun.

[B104] Gabrielsen OS, Hornes E, Korsnes L, Ruet A, Oyen TB (1989). Magnetic DNA affinity purification of yeast transcription factor τ – a new purification principle for the ultrarapid isolation of near homogeneous factor. Nucleic Acids Res.

[B105] Gabrielsen OS, Huet J (1993). Magnetic DNA affinity purification of yeast transcription factor. Meth Enzymol.

[B106] Hollung K, Gabrielsen OS, Jakobsen KS (1994). Enrichment of DNA-binding proteins from crude tissue for electrophoretic mobility shift assay using magnetic phospho cellulose particles. Nucleic Acids Res.

[B107] Risoen PA, Struksnes K, Myrset AH, Gabrielsen OS (1995). One-step magnetic purification of recombinant DNA-binding proteins using magnetizable phosphocellulose. Protein Expr Purif.

[B108] Zeng GC, Gao LY, Xia T, Tencomnao T, Yu RK (2003). Characterization of the 5'-flanking fragment of the human GM3-synthase gene. Biochim Biophys Acta.

[B109] Gershon PD, Moss B (1990). Early transcription factor subunits are encoded by vaccinia virus late genes. Proc Natl Acad Sci USA.

[B110] Ozyhar A, Gries M, Kiltz HH, Pongs O (1992). Magnetic DNA affinity purification of ecdysteroid receptor. J Steroid Biochem Mol Biol.

[B111] Kalivoda KA, Braun KW, Vimr ER (2003). Rapid purification of a prokaryotic regulatory protein with μMACS™ Streptavidin MicroBeads. MACS&more.

[B112] Rahat MA, Fry M (1993). Purification and characterization of p27, a protein from hepatocyte chromatin. Evidence suggesting that it binds selectively to guanine-rich single-stranded DNA. FEBS Lett.

[B113] Blank M, Weinschenk T, Priemer M, Schluesener H (2001). Systematic evolution of a DNA aptamer binding to rat brain tumor microvessels. Selective targeting of endothelial regulatory protein pigpen. J Biol Chem.

[B114] Albig A (2001). Isolation of mRNA binding proteins using the μMACS streptavidin kit. MACS&more.

[B115] McKay SJ, Cooke H (1992). A protein which specifically binds to single stranded TTAGGGn repeats. Nucleic Acids Res.

[B116] Chakraborty A, White SM, Schaefer TS, Ball ED, Dyer KF, Tweardy DJ (1996). Granulocyte colony-stimulating factor activation of Stat3a and Stat3β in immature normal and leukemic human myeloid cells. Blood.

[B117] Feghali CA, Wright TM (1995). Ligand-dependent and ligand-independent activation of the transcription factor γRf-1 in a cell-free system. Biochem J.

[B118] Ren LF, Chen H, Sternberg EA (1994). Tethered bandshift assay and affinity purification of a new DNA-binding protein. Biotechniques.

[B119] Allen TE, Heidmann S, Reed R, Myler PJ, Goringer HU, Stuart KD (1998). Association of guide RNA binding protein gBP21 with active RNA editing complexes in *Trypanosoma brucei*. Mol Cell Biol.

[B120] Honys D (2001). Isolation of proteins comprising native gene-specific messenger ribonucleoprotein particles using paramagnetic beads. Plant Sci.

[B121] Fantappie MR, Correa-Oliveira R, Caride EC, Geraldo EA, Agnew A, Rumjanek FD (1999). Comparison between site-specific DNA binding proteins of male and female *Schistosoma mansoni*. Comp Biochem Physiol B – Biochem Mol Biol.

[B122] Kelm RJ, Cogan JG, Elder PK, Strauch AR, Getz MJ (1999). Molecular interactions between single-stranded DNA-binding proteins associated with an essential MCAT element in the mouse smooth muscle alpha-actin promoter. J Biol Chem.

[B123] Daniels DA, Chen H, Hicke BJ, Swiderek KM, Gold L (2003). A tenascin-C aptamer identified by tumor cell SELEX: Systematic evolution of ligands by exponential enrichment. Proc Natl Acad Sci USA.

[B124] Skala-Rubinson H, Vinh J, Labas V, Kahn A, Tuy FPD (2002). Novel target sequences for Pax-6 in the brain-specific activating regions of the rat aldolase C gene. J Biol Chem.

[B125] Murphy MB, Fuller ST, Richardson PM, Doyle SA (2003). An improved method for the in vitro evolution of aptamers and applications in protein detection and purification. Nucleic Acids Res.

[B126] Tong XD, Sun Y (2001). Agar-based magnetic affinity support for protein adsorption. Biotechnol Progr.

[B127] Xue B, Tong XD, Sun Y (2001). Characterization of PVA-based magnetic affinity support for protein adsorption. Sep Sci Technol.

[B128] Xue B, Sun Y (2002). Fabrication and characterization of a rigid magnetic matrix for protein adsorption. J Chromatogr A.

[B129] Akgol S, Turkmen D, Denizli A (2004). Cu(II)-incorporated, histidine-containing, magnetic-metal-complexing beads as specific sorbents for the metal chelate affinity of albumin. J Appl Polym Sci.

[B130] Odabasi M, Uzun L, Denizli A (2004). Porous magnetic chelator support for albumin adsorption by immobilized metal affinity separation. J Appl Polym Sci.

[B131] Odabasi M, Denizli A (2004). Cibacron Blue F3GA-attached magnetic poly(2-hydroxyethyl methacrylate) beads for human serum albumin adsorption. Polym Int.

[B132] Ding XB, Sun ZH, Zhang WC, Peng YX, Wan GX, Jiang YY (2000). Adsorption/desorption of protein on magnetic particles covered by thermosensitive polymers. J Appl Polym Sci.

[B133] Ding XB, Sun ZH, Wan GX, Jiang YY (2000). Interactions between thermosensitive magnetic polymer microspheres and proteins. Acta Polym Sinica.

[B134] An XN, Su ZX, Zeng HM (2003). Preparation of highly magnetic chitosan particles and their use for affinity purification of enzymes. J Chem Technol Biotechnol.

[B135] Heeboll-Nielsen A, Dalkiaer M, Hubbuch JJ, Thomas ORT (2004). Superparamagnetic adsorbents for high-gradient magnetic fishing of lectins out of legume extracts. Biotechnol Bioeng.

[B136] Safarikova M, Safarik I (2000). One-step partial purification of *Solanum tuberosum *tuber lectin using magnetic chitosan particles. Biotechnol Lett.

[B137] Xu CJ, Xu KM, Gu HW, Zhong XF, Guo ZH, Zheng RK, Zhang XX, Xu B (2004). Nitrilotriacetic acid-modified magnetic nanoparticles as a general agent to bind histidine-tagged proteins. J Am Chem Soc.

[B138] Muller-Schulte D, Brunner H (1995). Novel magnetic microspheres on the basis of poly(vinyl alcohol) as affinity medium for quantitative detection of glycated hemoglobin. J Chromatogr A.

[B139] Dryden SC, Nahhas FA, Nowak JE, Goustin A-S, Tainsky MA (2003). Role for human SIRT2 NAD-dependent deacetylase activity in control of mitotic exit in the cell cycle. Mol Cell Biol.

[B140] Ohtsuki T, Sakurai M, Sato A, Watanabe K (2002). Characterization of the interaction between the nucleotide exchange factor EF-Ts from nematode mitochondria and elongation factor Tu. Nucleic Acids Res.

[B141] Suzuki M, Kamihira M, Shiraishi T, Takeuchi H, Kobayashi T (1995). Affinity partitioning of protein A using a magnetic aqueous two-phase system. J Ferment Bioeng.

[B142] Liabakk NB, Nustad K, Espevik T (1990). A rapid and sensitive immunoassay for tumor necrosis factor using magnetic monodisperse polymer particles. J Immunol Methods.

[B143] Zhang ZR, O'Sullivan DA, Lyddiatt A (1999). Magnetically stabilised fluidised bed adsorption: practical benefit of uncoupling bed expansion from fluid velocities in the purification of a recombinant protein from *Escherichia coli*. J Chem Technol Biotechnol.

[B144] Simonsen A, Stang E, Bremnes B, Roe M, Prydz K, Bakke O (1997). Sorting of MHC class II molecules and the associated invariant chain (Ii) in polarized MDCK cells. J Cell Sci.

[B145] Mikaelsdottir EK, Valgeirsdottir S, Eyfjord JE, Rafnar T (2004). The Icelandic founder mutation BRCA2 999del5: analysis of expression. Breast Cancer Res.

[B146] Welter BH, Price TM (1999). Magnetic separation to concentrate the estrogen receptor from adipose tissue for western analysis. Biotechniques.

[B147] Chen-Cleland TA, Boffa LC, Carpaneto EM, Mariani MR, Valentin E, Mendez E, Allfrey VG (1993). Recovery of transcriptionally active chromatin restriction fragments by binding to organomercurial-agarose magnetic beads. A rapid and sensitive method for monitoring changes in higher order chromatin structure during gene activation and repression. J Biol Chem.

[B148] Knutson VP, Donnelly PV, Balba Y, Lopez-Reyes M (1995). Insulin resistance is mediated by a proteolytic fragment of the insulin receptor. J Biol Chem.

[B149] Chakraborty A, Dyer KF, Cascio M, Mietzner TA, Tweardy DJ (1999). Identification of a novel Stat3 recruitment and activation motif within the granulocyte colony-stimulating factor receptor. Blood.

[B150] Tanaka T, Matsunaga T (2001). Detection of HbA(1c) by boronate affinity immunoassay using bacterial magnetic particles. Biosens Bioelectron.

[B151] Karlsson GB, Platt FM (1991). Analysis and isolation of human transferrin receptor using the OKT-9 monoclonal antibody covalently cross-linked to magnetic beads. Anal Biochem.

[B152] Apoga D, Ek B, Tunlid A (2001). Analysis of proteins in the extracellular matrix of the plant pathogenic fungus *Bipolaris sorokiniana *using 2-D gel electrophoresis and MS/MS. FEMS Microbiol Lett.

[B153] Hincha DK, Neukamm B, Sror HAM, Sieg F, Weckwarth W, Ruckels M, Lullien-Pellerin V, Schroder W, Schmitt JM (2001). Cabbage cryoprotectin is a member of the nonspecific plant lipid transfer protein gene family. Plant Physiol.

[B154] Peter J, Unverzagt C, Lenz H, Hoesel W (1999). Purification of prostate-specific antigen from human serum by indirect immunosorption and elution with a hapten. Anal Biochem.

[B155] Peter J, Unverzagt C, Krogh TN, Vorm O, Hoesel W (2001). Identification of precursor forms of free prostate-specific antigen in serum of prostate cancer patients by immunosorption and mass spectrometry. Cancer Res.

[B156] Yang Y-S, Yang M-CW, Wang B, Weissler JC (2001). BR22, a novel protein, interacts with thyroid transcription factor-1 and activates the human surfactant protein B promoter. Am J Respir Cell Mol Biol.

[B157] Persaud DR, Yousefi V, Haunerland N (2003). Efficient isolation, purification, and characterization of the *Helicoverpa zea *VHDL receptor. Protein Expr Purif.

[B158] Salazar R, Brandt R, Kellermann J, Krantz S (2000). Purification and characterization of a 200 kDa fructosyllysine-specific binding protein from cell membranes of U937 cells. Glycoconjugate J.

[B159] Girault S, Chassaing G, Blais JC, Brunot A, Bolbach G (1996). Coupling of MALDI-TOF mass analysis to the separation of biotinylated peptides by magnetic streptavidin beads. Anal Chem.

[B160] Ji Z, Pinon DI, Miller LJ (1996). Development of magnetic beads for rapid and efficient metal-chelate affinity purifications. Anal Biochem.

[B161] Samson I, Rozenski J, Vanaerschot A, Samyn B, Vanbeeumen J, Herdewijn P (1995). Screening of a synthetic pentapeptide library composed of D-amino acids against fructose-1,6-biphosphate aldolase. Lett Pept Sci.

[B162] Seino S, Kinoshita T, Otome Y, Nakagawa T, Okitsu K, Nakayama T, Sekino T, Niihara K, Yamamoto TA (2004). Radiation-induced synthesis of Au/Fe oxide nanocomposite particles for magnetic separation of biomolecules. J Ceram Process Res.

[B163] Prioult G, Turcotte C, Labarre L, Lacroix C, Fliss I (2000). Rapid purification of nisin Z using specific monoclonal antibody-coated magnetic beads. Int Dairy J.

